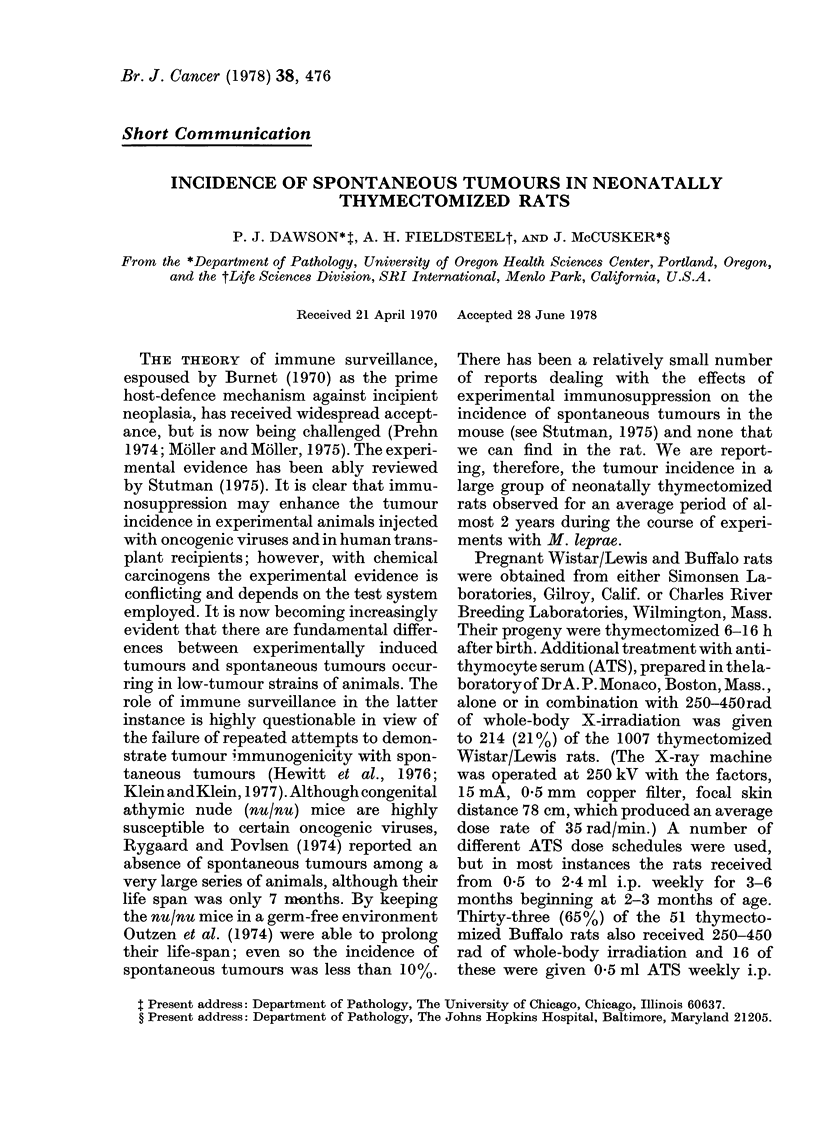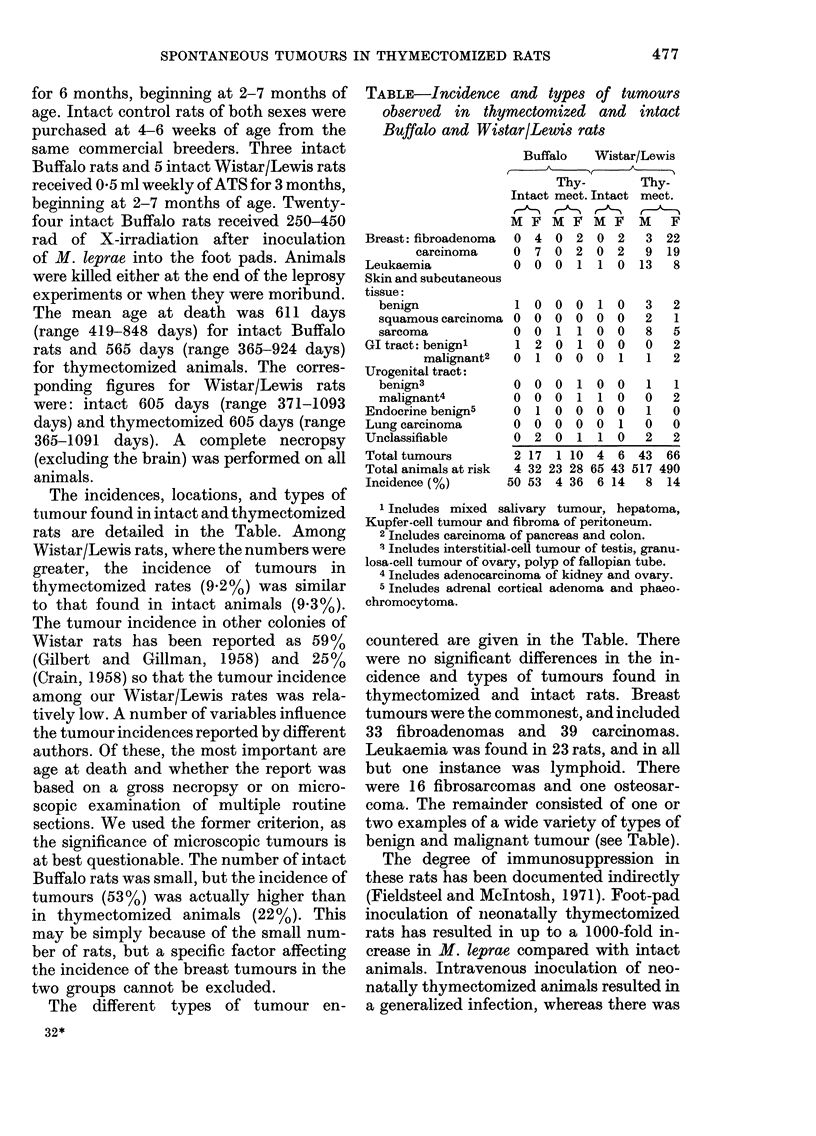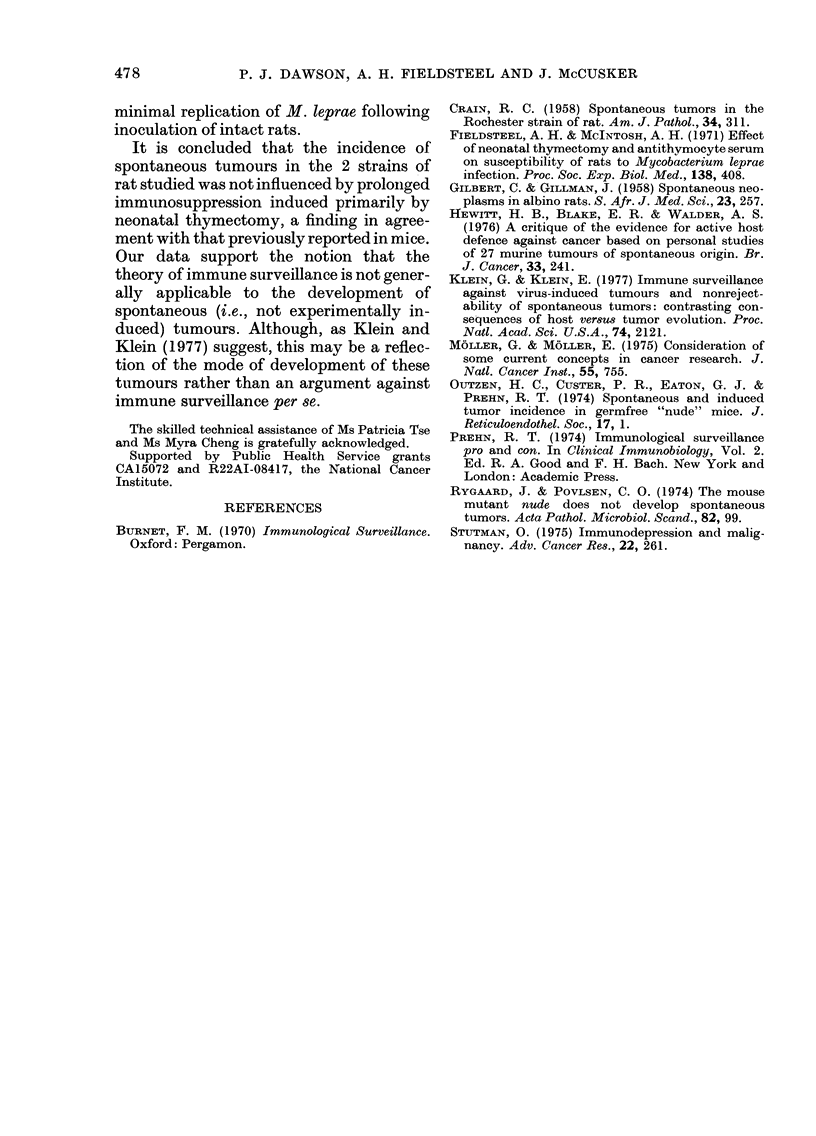# Incidence of spontaneous tumours in neonatally thymectomized rats.

**DOI:** 10.1038/bjc.1978.234

**Published:** 1978-09

**Authors:** P. J. Dawson, A. H. Fieldsteel, J. McCusker


*Br. J. Cancer* (1978) **38**, 476

***Short Communication***

### INCIDENCE OF SPONTANEOUS TUMOURS IN NEONATALLY THYMECTOMIZED RATS

P. J. DAWSON*‡, A. H. FIELDSTEEL†, AND J. McCUSKER*§

*From the *Department of Pathology, University of Oregon Health Sciences Center, Portland, Oregon, and the †Life Sciences Division, SRI International, Menlo Park, California, U.S.A.*

Received 21 April 1970 Accepted 28 June 1978

THE THEORY of immune surveillance, espoused by Burnet (1970) as the prime host-defence mechanism against incipient neoplasia, has received widespread acceptance, but is now being challenged (Prehn 1974; Möller and Möller, 1975). The experimental evidence has been ably reviewed by Stutman (1975). It is clear that immunosuppression may enhance the tumour incidence in experimental animals injected with oncogenic viruses and in human transplant recipients; however, with chemical carcinogens the experimental evidence is conflicting and depends on the test system employed. It is now becoming increasingly evident that there are fundamental differences between experimentally induced tumours and spontaneous tumours occurring in low-tumour strains of animals. The role of immune surveillance in the latter instance is highly questionable in view of the failure of repeated attempts to demonstrate tumour immunogenicity with spontaneous tumours (Hewitt *et al.*, 1976; Klein and Klein, 1977). Although congenital athymic nude (*nu/nu*) mice are highly susceptible to certain oncogenic viruses, Rygaard and Povlsen (1974) reported an absence of spontaneous tumours among a very large series of animals, although their life span was only 7 months. By keeping the *nu/nu* mice in a germ-free environment Outzen *et al.* (1974) were able to prolong their life-span; even so the incidence of spontaneous tumours was less than 10%. There has been a relatively small number of reports dealing with the effects of experimental immunosuppression on the incidence of spontaneous tumours in the mouse (see Stutman, 1975) and none that we can find in the rat. We are reporting, therefore, the tumour incidence in a large group of neonatally thymectomized rats observed for an average period of almost 2 years during the course of experiments with *M. leprae*.

Pregnant Wistar/Lewis and Buffalo rats were obtained from either Simonsen Laboratories, Gilroy, Calif. or Charles River Breeding Laboratories, Wilmington, Mass. Their progeny were thymectomized 6–16 h after birth. Additional treatment with antithymocyte serum (ATS), prepared in the laboratory of Dr A. P. Monaco, Boston, Mass., alone or in combination with 250–450 rad of whole-body X-irradiation was given to 214 (21%) of the 1007 thymectomized Wistar/Lewis rats. (The X-ray machine was operated at 250 kV with the factors, 15 mA, 0·5 mm copper filter, focal skin distance 78 cm, which produced an average dose rate of 35 rad/min.) A number of different ATS dose schedules were used, but in most instances the rats received from 0·5 to 2·4 ml i.p. weekly for 3–6 months beginning at 2–3 months of age. Thirty-three (65%) of the 51 thymectomized Buffalo rats also received 250–450 rad of whole-body irradiation and 16 of these were given 0·5 ml ATS weekly i.p.

‡ Present address: Department of Pathology, The University of Chicago, Chicago, Illinois 60637.
§ Present address: Department of Pathology, The Johns Hopkins Hospital, Baltimore, Maryland 21205.

for 6 months, beginning at 2–7 months of age. Intact control rats of both sexes were purchased at 4–6 weeks of age from the same commercial breeders. Three intact Buffalo rats and 5 intact Wistar/Lewis rats received 0·5 ml weekly of ATS for 3 months, beginning at 2–7 months of age. Twenty-four intact Buffalo rats received 250–450 rad of X-irradiation after inoculation of *M. leprae* into the foot pads. Animals were killed either at the end of the leprosy experiments or when they were moribund. The mean age at death was 611 days (range 419–848 days) for intact Buffalo rats and 565 days (range 365–924 days) for thymectomized animals. The corresponding figures for Wistar/Lewis rats were: intact 605 days (range 371–1093 days) and thymectomized 605 days (range 365–1091 days). A complete necropsy (excluding the brain) was performed on all animals.

The incidences, locations, and types of tumour found in intact and thymectomized rats are detailed in the Table. Among Wistar/Lewis rats, where the numbers were greater, the incidence of tumours in thymectomized rates (9·2%) was similar to that found in intact animals (9·3%). The tumour incidence in other colonies of Wistar rats has been reported as 59% (Gilbert and Gillman, 1958) and 25% (Crain, 1958) so that the tumour incidence among our Wistar/Lewis rates was relatively low. A number of variables influence the tumour incidences reported by different authors. Of these, the most important are age at death and whether the report was based on a gross necropsy or on microscopic examination of multiple routine sections. We used the former criterion, as the significance of microscopic tumours is at best questionable. The number of intact Buffalo rats was small, but the incidence of tumours (53%) was actually higher than in thymectomized animals (22%). This may be simply because of the small number of rats, but a specific factor affecting the incidence of the breast tumours in the two groups cannot be excluded.

TABLE—*Incidence and types of tumours observed in thymectomized and intact Buffalo and Wistar/Lewis rats*

| | Buffalo | | | | Wistar/Lewis | | | |
|---|---|---|---|---|---|---|---|---|
| | Intact | | Thymect. | | Intact | | Thymect. | |
| | M | F | M | F | M | F | M | F |
| Breast: fibroadenoma | 0 | 4 | 0 | 2 | 0 | 2 | 3 | 22 |
| carcinoma | 0 | 7 | 0 | 2 | 0 | 2 | 9 | 19 |
| Leukaemia | 0 | 0 | 0 | 1 | 1 | 0 | 13 | 8 |
| Skin and subcutaneous tissue: | | | | | | | | |
| benign | 1 | 0 | 0 | 0 | 1 | 0 | 3 | 2 |
| squamous carcinoma | 0 | 0 | 0 | 0 | 0 | 0 | 2 | 1 |
| sarcoma | 0 | 0 | 1 | 1 | 0 | 0 | 8 | 5 |
| GI tract: benign[1] | 1 | 2 | 0 | 1 | 0 | 0 | 0 | 2 |
| malignant[2] | 0 | 1 | 0 | 0 | 0 | 1 | 1 | 2 |
| Urogenital tract: | | | | | | | | |
| benign[3] | 0 | 0 | 0 | 1 | 0 | 0 | 1 | 1 |
| malignant[4] | 0 | 0 | 0 | 1 | 1 | 0 | 0 | 2 |
| Endocrine benign[5] | 0 | 1 | 0 | 0 | 0 | 0 | 1 | 0 |
| Lung carcinoma | 0 | 0 | 0 | 0 | 0 | 1 | 0 | 0 |
| Unclassifiable | 0 | 2 | 0 | 1 | 1 | 0 | 2 | 2 |
| Total tumours | 2 | 17 | 1 | 10 | 4 | 6 | 43 | 66 |
| Total animals at risk | 4 | 32 | 23 | 28 | 65 | 43 | 517 | 490 |
| Incidence (%) | 50 | 53 | 4 | 36 | 6 | 14 | 8 | 14 |

[1] Includes mixed salivary tumour, hepatoma, Kupfer-cell tumour and fibroma of peritoneum.
[2] Includes carcinoma of pancreas and colon.
[3] Includes interstitial-cell tumour of testis, granulosa-cell tumour of ovary, polyp of fallopian tube.
[4] Includes adenocarcinoma of kidney and ovary.
[5] Includes adrenal cortical adenoma and phaeochromocytoma.

The different types of tumour encountered are given in the Table. There were no significant differences in the incidence and types of tumours found in thymectomized and intact rats. Breast tumours were the commonest, and included 33 fibroadenomas and 39 carcinomas. Leukaemia was found in 23 rats, and in all but one instance was lymphoid. There were 16 fibrosarcomas and one osteosarcoma. The remainder consisted of one or two examples of a wide variety of types of benign and malignant tumour (see Table).

The degree of immunosuppression in these rats has been documented indirectly (Fieldsteel and McIntosh, 1971). Foot-pad inoculation of neonatally thymectomized rats has resulted in up to a 1000-fold increase in *M. leprae* compared with intact animals. Intravenous inoculation of neonatally thymectomized animals resulted in a generalized infection, whereas there was minimal replication of *M. leprae* following inoculation of intact rats.

It is concluded that the incidence of spontaneous tumours in the 2 strains of rat studied was not influenced by prolonged immunosuppression induced primarily by neonatal thymectomy, a finding in agreement with that previously reported in mice. Our data support the notion that the theory of immune surveillance is not generally applicable to the development of spontaneous (*i.e.*, not experimentally induced) tumours. Although, as Klein and Klein (1977) suggest, this may be a reflection of the mode of development of these tumours rather than an argument against immune surveillance *per se*.

The skilled technical assistance of Ms Patricia Tse and Ms Myra Cheng is gratefully acknowledged.

Supported by Public Health Service grants CA15072 and R22AI-08417, the National Cancer Institute.

### REFERENCES

BURNET, F. M. (1970) *Immunological Surveillance.* Oxford: Pergamon.

CRAIN, R. C. (1958) Spontaneous tumors in the Rochester strain of rat. *Am. J. Pathol.*, **34,** 311.

FIELDSTEEL, A. H. & McINTOSH, A. H. (1971) Effect of neonatal thymectomy and antithymocyte serum on susceptibility of rats to *Mycobacterium leprae* infection. *Proc. Soc. Exp. Biol. Med.*, **138,** 408.

GILBERT, C. & GILLMAN, J. (1958) Spontaneous neoplasms in albino rats. *S. Afr. J. Med. Sci.*, **23,** 257.

HEWITT, H. B., BLAKE, E. R. & WALDER, A. S. (1976) A critique of the evidence for active host defence against cancer based on personal studies of 27 murine tumours of spontaneous origin. *Br. J. Cancer*, **33,** 241.

KLEIN, G. & KLEIN, E. (1977) Immune surveillance against virus-induced tumours and nonrejectability of spontaneous tumors: contrasting consequences of host *versus* tumor evolution. *Proc. Natl. Acad. Sci. U.S.A.*, **74,** 2121.

MÖLLER, G. & MÖLLER, E. (1975) Consideration of some current concepts in cancer research. *J. Natl. Cancer Inst.*, **55,** 755.

OUTZEN, H. C., CUSTER, P. R., EATON, G. J. & PREHN, R. T. (1974) Spontaneous and induced tumor incidence in germfree "nude" mice. *J. Reticuloendothel. Soc.*, **17,** 1.

PREHN, R. T. (1974) Immunological surveillance *pro* and *con.* In *Clinical Immunobiology*, Vol. 2. Ed. R. A. Good and F. H. Bach. New York and London: Academic Press.

RYGAARD, J. & POVLSEN, C. O. (1974) The mouse mutant *nude* does not develop spontaneous tumors. *Acta Pathol. Microbiol. Scand.*, **82,** 99.

STUTMAN, O. (1975) Immunodepression and malignancy. *Adv. Cancer Res.*, **22,** 261.